# Ageing, exposure to pollution, and interactions between climate change and local seasons as oxidant conditions predicting incident hematologic malignancy at KINSHASA University clinics, Democratic Republic of CONGO (DRC)

**DOI:** 10.1186/s12885-017-3547-3

**Published:** 2017-08-23

**Authors:** Mireille Solange Nganga Nkanga, Benjamin Longo-Mbenza, Oladele Vincent Adeniyi, Jacques Bikaula Ngwidiwo, Antoine Lufimbo Katawandja, Paul Roger Beia Kazadi, Alain Nganga Nzonzila

**Affiliations:** 1 Département de Biologie Médicale, Service de Biologie Clinique, CUK, Faculté de Médecine, Kinshasa, Democratic Republic of Congo; 2 Centre Hospitalier OVD, Kinshasa, Democratic Republic of Congo; 3 Hôpital Saint- Joseph, Kinshasa, Democratic Republic of Congo; 4 Faculty of Health Sciences, Walter Sisulu University, Private Bag X1, 5117 Mthatha, South Africa; 5 Cecilia Makiwane Hospital/Walter Sisulu University, Faculty of Health Sciences, East London, South Africa

**Keywords:** Environmental epidemiology, Hematologic malignancies, Aging, Central Africa

## Abstract

**Background:**

The global burden of hematologic malignancy (HM) is rapidly rising with aging, exposure to polluted environments, and global and local climate variability all being well-established conditions of oxidative stress. However, there is currently no information on the extent and predictors of HM at Kinshasa University Clinics (KUC), DR Congo (DRC). This study evaluated the impact of bio-clinical factors, exposure to polluted environments, and interactions between global climate changes (EL Nino and La Nina) and local climate (dry and rainy seasons) on the incidence of HM.

**Methods:**

This hospital-based prospective cohort study was conducted at Kinshasa University Clinics in DR Congo. A total of 105 black African adult patients with anaemia between 2009 and 2016 were included. HM was confirmed by morphological typing according to the French-American-British (FAB) Classification System. Gender, age, exposure to traffic pollution and garages/stations, global climate variability (El Nino and La Nina), and local climate (dry and rainy seasons) were potential independent variables to predict incident HM using Cox regression analysis and Kaplan Meier curves.

**Results:**

Out of the total 105 patients, 63 experienced incident HM, with an incidence rate of 60%. After adjusting for gender, HIV/AIDS, and other bio-clinical factors, the most significant independent predictors of HM were age ≥ 55 years (HR = 2.4; 95% CI 1.4–4.3; *P* = 0.003), exposure to pollution and garages or stations (HR = 4.9; 95% CI 2–12.1; *P* < 0.001), combined local dry season + La Nina (HR = 4.6; 95%CI 1.8–11.8; *P* < 0.001), and combined local dry season + El Nino (HR = 4; 95% CI 1.6–9.7; *P* = 0.004). HM types included acute myeloid leukaemia (28.6% *n* = 18), multiple myeloma (22.2% *n* = 14), myelodysplastic syndromes (15.9% *n* = 10), chronic myeloid leukaemia (15.9% *n* = 10), chronic lymphoid leukaemia (9.5% *n* = 6), and acute lymphoid leukaemia (7.9% *n* = 5). After adjusting for confounders using Cox regression analysis, age ≥ 55 years, exposure to pollution, combined local dry season + La Nina and combined local dry season + El Nino were the most significant predictors of incident hematologic malignancy.

**Conclusion:**

These findings highlight the importance of aging, pollution, the dry season, El Nino and La Nina as related to global warming as determinants of hematologic malignancies among African patients from Kinshasa, DR Congo. Cancer registries in DRC and other African countries will provide more robust database for future researches on haematological malignancies in the region.

## Background

Cancer constitutes a greater healthcare burden in developed countries than in less developed countries [1]. The occurrence of cancer is, however, increasing worldwide because of the growth and aging of the world population [1–6]. The increasing prevalence of established risk factors such as exposure to polluted environments, the interaction of global climate with local climate conditions, oxidative stress, rapid urbanization and economic transition are also contributory factors [3].

Hematologic malignancies (HM) are a leading cause of morbidity, co-morbidity, mortality, and disability in both rich and resource-poor countries such as Democratic Republic of Congo (DRC) [1, 5, 7–12]. The incidence of HM is associated with aging, exposure to pollution (in residence or in workplace) and endemic infections (HIV and other viruses) [13, 14]. There is no comprehensive hypothesis regarding the manner in which these risk factors act.

DRC in general and its capital Kinshasa in particular are experiencing epidemiologic and nutritional transitions which are responsible for the increase in non-communicable diseases (NCD) such as cardiovascular diseases, Type-2 diabetes mellitus (T2DM) and stroke. All of these are related to oxidative stress [15], aging and climate variability (El Nino-La Nina [16] and HM [8].

However, there has been no research at Kinshasa University Clinics (KUCs) or in DRC generally to improve our understanding of the roles of personal attributes, socioeconomic factors and physical environments in the emerging epidemic of HM in Kinshasa Province, a region experiencing many socio-economic and political crises. For this study, the researchers hypothesized that factors such as aging, pollution, hypoxic environment, climate change and cold seasons are all oxidant conditions increasing the vulnerability of patients to HM in KUCs.

Therefore, this study evaluated the impact of bio-clinical factors, exposure to pollutants and interactions between global climate change (El Nino-La Nina) and local seasons on HM incidence.

## Methods

### Study design

This study was a KUC-based prospective study involving 105 black African adult patients with anaemia between 2009 and 2016.

### Sampling

The study population comprised anaemic black African patients according to pre-defined inclusion and exclusion criteria. This eligible population was characterized by chronic anaemia with episodic fever and fatigue.

### Sample size

$$ N=4{\left({\mathrm{Z}}_{\upalpha}\right)}^2\mathrm{P}\left(1\hbox{-} \mathrm{P}\right)/{\mathrm{W}}^2 $$

Where N is the sample size, Z_α_ the Z-value for a two sided α of 0.05 and therefore a confidence interval of 95%, P is the proportion of people with chronic anaemia expected to present with incident HM. Since the proportion of people with chronic anaemia who present with incident HM in the study population was not known, the authors assumed a proportion of 0.5 which will give the maximum sample size estimate [17], and a width or acceptable error (W) of 0.2 (i.e. an incidence of 0.5 ± 0.2). For a two sided α of 0.05 and therefore a confidence interval of 95%, z_α_ = 1.96.

So the sample size *N* = 4(1.96)^2^(0.5 × 0.5) / 0.04.

*N* = 96 which was approximated to 100.

Allowance for missing data was made: 100 + 20% of potential misses = 120.

Inclusion criteria were age ≥ 20 years and myelogram-diagnosed HM. Patients with any myelogram result other than HM and those patients who did not wish to participate in the study were excluded.

### Laboratory data

To define structural (morphological) markers for diagnosing HM, 3.5 ml of blood were obtained by venepuncture in tubes with anticoagulant ethylenediaminetetraacetic acid (EDTA) for the hemogram. In addition, three smears of peripheral blood were performed. A sample taken from a tube with 3.8% citrate was used for the determination of the erythrocyte sedimentation rate (ESR). The bone marrow aspiration was performed either at the level of the breastbone or at the level of the posterior iliac spine to collect 0.5 ml of medullary content. Ten slides were displayed for the morphological study using the May Grünwald Giemsa (MGG) stains and for the special stains (Sudan black B, Periodic Acid Shift, Coloring of Perls). Medullary smears were also obtained for colored staining using the MGG viewed under the multi-ordinary microscope typical of Olympus.

### Potential predictors

Independent variables were age, sex, infectious syndrome (HIV/AIDS, sepsis, and bacteremia), blood transfusion count, levels of hematologic markers (hemoglobin, white cell count and platelets), exposure to pollution, global climate variability (El Nino-La Nina), and local climate (seasons).

Information on gender, age, exposure to traffic pollution and garages or stations, global climate variability (El Nino and La Nina), and local climate (dry and rainy seasons) was obtained. Climate changes caused by global warming conditions caused climate variability, which was defined as short-term fluctuations around the mean climate state. Climate variability also refers to changes in climate patterns such as precipitation, weather conditions, temperature and humidity [16]. El Nino Southern Oscillation is associated with climate changes in the tropical and sub-tropical regions as a result of temperature anomalies from the warming and cooling of the ocean surface [18, 19].

El Nino can be defined as warmer-than-normal sea or ocean surface temperatures [16] and La Nina refers to cooler-than-normal sea or ocean surface temperatures [16]. El Nino years (2009, 2010, 2013 and 2015) and La Nina years (2011, 2012 and 2014) were defined by the THI Oceanic Nino Index (ONI). (http://www.cpc.ncep.noaa.gov/products/analysis_monitoring/ensostuff/ensoyears.shtml).

Local climates and seasons were defined by meteorological parameters (monthly temperatures, humidity, winds, fog, and precipitation). The dry season (very dry for June to September and less dry for January to March), cold months and rainy periods (high rainfall for October to December, lower rainfall for April and May) characterized the local climate. The interaction between global climate variability and local climate typically followed the pattern of: local dry season + global La Nina; local dry season + global El Nino; local rainy season + global La Nina; and local rainy season + global El Nino.

Places of residence and/or occupation close to high-traffic-volume roads, stations/garages, sources of dust, smoke or industrial pollutants were classified as polluted environments.

The clinical variables were infectious syndromes (bacteraemia, sepsis), bone pain, fever, splenomegaly and fatigue.

### Dependent variable

The incidence of HM and each of its subtypes was confirmed by morphological typing according to the French-American-British (FAB) WHO classification system (Mounia, WHO).

### Statistical analysis

The reliability and validity of this research relies on the accuracy of data and the consistency of the tools and procedures used—the research design. In order to achieve the highest standard of accuracy, the researchers avoided confounding factors and foreseeable information bias and selection bias.

In a univariate analysis, continuous variables were symmetrical and expressed as means ± standard deviation (SD), compared between two groups (HM and patients with anaemia but having a normal myelogram) using the Student’s t-test. However, categorical variables were presented as frequencies (n = number) and prevalence (%), comparing the two groups using the chi-square test. The researchers performed logistic regression and the Cox regression model to calculate adjusted multivariate hazard ratios (HR = beta exponential) for the risk of incident HM with their corresponding 95% confidence interval (95% CI). Kaplan-Meir curves after the Cox regression model produced a one minus cumulative survival function with a log-rank test of the equality of survival distribution and time medians for the different levels of stratified covariates.

As age and laboratory optimal cut-off points were unknown, the effective (accurate and sensitive) cut-off values for discriminating HM and normal myelogram were tested a posteriori, using the Receiver Operating Characteristic curves (ROC) method. The area under the curve (AUC for c-static) was calculated with its corresponding Standard Error (SE), 95% CI, and *P*-value. The criterion for two-sided statistical significance was *p*-value <0.05. All analyses were performed using SPSS software version 23.0 for Windows (IBM/SPSS Inc., New York, USA).

## Results

Of the total number of patients (*n* = 105), 53.3% (*n* = 56) were males and 46.7% (*n* = 49) were females, giving a sex ratio of almost 1:1. The incidence of HM in all patients was 60% (*n* = 63); with a slightly higher incidence in males than in females (55.6%, *n* = 35 for men vs. 44.4%, *n* = 28 for women). In the normal myelogram group, the figures were 50% *n* = 21 for men vs. 50% *n* = 21 for women), without any significant impact of sex (*P* = 0.576) on HM incidence.

In considering incidences (*P* = 0.161) across HM subtypes, the sex ratios of men to women were different, as follows: in myelodysplastic syndrome (MDS), the sex ratio of men to women was 4:1 (8 men vs. 2 women). In multiple myeloma (MM), the sex ratio of men to women was 3:1 (10 men vs. 4 women). In acute myeloid leukaemia (AML), the sex ratio of men to women was 1: 1 (10 man vs. 8 women). In chronic myeloid leukaemia (CML), the sex ratio of men to women was 1:1 (4 men vs. 6 women). In acute lymphoid leukaemia (ALL), the sex ratio of men to women was 1:1 (2 men vs. 3 women) and in normal myelogram results the sex ratio was 1:1 (21 men vs. 21 women).

In chronic lymphoid leukaemia (CLL), the sex ratio was paradoxically 5 women: 1 man (5 women vs. 1 man). In the participants with incident HM, higher age, blood transfusion count and white cell count, but lower levels of haemoglobin and platelet counts, were observed as significant (*P* < 0.005), more than in patients with normal myelogram results (Table 1). In incident HM patients, Table 2 shows significant associations between aging (yes ≥50 years), HIV/AIDS (yes), bacteraemia (yes) and incident HM.

**Table 1:** Comparison of mean values of age, blood transfusion, and hematologic parameters between HM incident (*n* = 63) and normal myelogram (*n* = 42)

Variables	HM incidence Mean ± SD	Normal myelogram Mean ± SD	*p*-value
Age (years)	61 ± 16.5	39.5 ± 13.8	<0.0001
Blood transfusion (number)	9.6 ± 3.6	2.2 ± 1.1	<0.0001
Hemoglobin (g/dL)	6.8 ± 2.5	8.2 ± 2.6	0.005
White cell count (mm^3^)	22,936 ± 22,244	7029 ± 4053	<0.0001
Platelet (mm^3^)	189,395 ± 118,948	249,107 ± 151,751	0.026

*SD* standard deviation

**Table 2:** Univariate association between aging, sepsis, HIV/AIDS, bacteraemia, and HM Incidence

Variables	HM incidence % (n)	*p*-value
Age		
Yes ≥ 50 years	79.7 (47/59)	<0.0001
No < 50 years	34.8 (16/46)	
Sepsis		
Yes	37.9 (11/29)	<0.0001
No	68.4 (52/76)	
HIV/AIDS		
Yes	28.6 (18/63)	0.002
No	4.8 (2/42)	
Bacteremia		
Yes	75 (45/60)	<0.0001
No	40 (18/45)	

*HM* haematological malignancy

The prevalence of HIV/AIDS varied significantly (*P* < 0.0001) across HM subtypes, as follows: The highest rate was in MM (*n* = 8/14), intermediate rates were in 2/5 of ALL, and rates of 2/6 were found in CML. The lowest rates of HIV/AIDS were 3/18 for AML, 1/10 for MDS and 2/42 for normal myelogram. The trends of incident HM peaked in El Nino 2009 and the lowest incidence was seen in La Nina 2014. The peak incidence of HM was observed between June to August, the very dry season. The majority of patients with HM were diagnosed during the dry and cold season. However, there was no significant difference in HM incidence by global climate variability.

In univariate analysis, the local dry and cold season, both the La Nina/cold season and El Nino/warm season, combined local dry season + global La Nina, and exposure to pollution were significantly associated with HM incidence (Table 3).

**Table 3:** Univariate association between local climate, global climate, combined global and local climate, exposure to pollution, and HM incidence

Variables	HM incidence	*p*-value
Local climate		
Dry cold season	83.9 (52/62)	<0.0001
Rainy and hot season	25.6 (11/43)	
Global climate variability		
La Nina and cold	72.1 (31/43)	0.035
El Nino and warm	51.6 (32/62)	0.035
Combined global and local climate		
Local dry season + global La Nina	86.7 (26/30)	<0.0001
Local dry season + global El Nino	81.2 (26/32)	
Local rainy season + global La Nina	32.5 (5/13)	
Local rainy season + global El Nino	20 (6/30)	
Exposure to pollution		
Yes	83.8 (57/60)	<0.0001
No	16.2 (6/37)	

Among all markers of HM, only the mean values of platelets varied significantly across combined global and local climates. The lowest levels of HM were observed in the local dry season + global La Nina and the highest levels were observed in the local rainy season + global El Nino.

After adjusting for mean values of platelets, sepsis, HIV/AIDS, and bacteraemia, using Cox’s regression analysis, the significant independent predictors of incident HM were: exposure to pollution, with a six-fold risk increase; combined local dry season + global La Nina, with a three-fold risk increase; combined local rainy season + global El Nino, with a three-fold risk increase; combined local dry season + global La Nina, with a two-fold risk increase; and aging with a two-fold risk increase (Table 4).

**Table 4:** Independent predictors of HM incidence using multivariate Cox’s Regression analysis

	B	SE	Wald	df	Sig.	HR	95.0% CI for HR	
							Lower	Upper
Exposure 1	1.714	.453	14.308	1	.000	5.550	2.284	13.488
Combined Climates (1)	1.249	.481	6.742	1	.009	3.488	1.358	8.955
Combined Climates (2)	1.099	.476	5.334	1	.021	3.001	1.181	7.626
Combined Climates (3)	.501	.610	.676	1	.411	1.651	.500	5.453
Aging 1	.619	.293	4.476	1	.034	1.857	1.047	3.295

*SE* standard error, *HR* hazard ratio

Exposure1 = exposure to pollution

Combined climates (1) = combined local dry season and global Lanina

Combined climates (2) = combined local dry season and global Elnino

Combined climates (3) = combined local rainy season and global Lanina

## Discussion

This is the first time the factors of aging, sex, infectious syndrome, traditional hematologic markers, exposure to pollution (residence or occupation close to traffic, stations/garages, dust, smoke, or industrial areas), local seasons and climates, global climate variability and the interaction between global and local climates were tested as oxidant conditions for emerging HM at KUC, Kinshasa Province, DRC. Therefore, univariate analysis and a multivariate Cox’s regression model were used to understand the pathophysiology and the environmental epidemiology (ecology) of HM incidence in a prospective cohort for anaemic patients managed at KUC. The present findings defined potential predictors and biomarkers as well as risk factors of incident HM.

### Descriptive characteristics

The present study confirmed an emerging burden of HM incidence among anaemic patients with an estimated incidence rate of 60%. This magnitude was almost eight-fold higher than the HM incidence of 8.7% reported for sub-Saharan Africa in 2008 [1, 20]. The present Congolese epidemic of HM (an incidence rate of 60% in 2016) was already close to the HM incidence rate projected to be 75% by 2030 for all of sub-Saharan Africa [1, 7]. Among the patients with incident HM (*n* = 63), this study confirmed AML and MM as the most prevalent malignancies, which is similar to previous reports from Kinshasa, DRC and other parts of Africa [1, 6, 11].

### Potential risk factors for HM

In this study, aging, sepsis, bacteraemia, HIV/AIDS, hematologic markers, exposure to pollution, local climate, global climate variability and combined global and local climate were identified as potential univariate predictors of HM incidence. As reported in the literature [21, 22] and by the present study, sex (sex ratio = 1) was not significantly associated with AML, with CML or with ALL. However, male sex or female sex were significantly associated with other HM subtypes, as follows: male predominance in the MM and MDS subtypes and female predominance in the CLL subtype.

### HIV/aids

As has been reported in both developed and poor countries [1, 23–32], a univariate significant association exists between HM and HIV for patients both taking and not taking antiretroviral therapy. In sub–Saharan Africa, including DR Congo, HIV infection (well established as an oxidative stress condition [15]), the Epstein–Bar virus (EBV), the Kaposi sarcoma–associated herpes virus (KSHV), and malaria (a condition of endothelial dysfunction, a risk factor of HM oxidative stress [32]) are all identified as significant ecological risk factors of HM [1, 33–37].

### Independent predictors of HM incidence

In this study using multivariate Cox’s regression analysis, only exposure to pollution, combined local dry season + global La Nina climate variability, combined local dry season + global El Nino climate variability, and age ≥ 50 years were identified as the most significant and independent predictors of HM-incidence among anaemic black Central Africans. Climate change leads to over-exposure to ultraviolet rays and lower levels of vitamin D, resulting in decreased levels of immunity. In addition, chronic exposure to benzene products, lead and mercury in the environment increases the risk of HM. Climate change, environmental pollution, aging and anaemic hypoxia are associated with an imbalance in the anti-oxidant/oxidant state (oxidative stress), which increases the risk of HM incidence [13, 21, 38–46].

Aging in particular is associated with the pathogenesis of clonal myeloid diseases such as chronic myeloid leukaemia [47]. In Kinshasa, the early warning system impacts local seasons, climate variability (La Nina and El Nino) and ischemic stroke morbidity and mortality [16].

### Clinical implications and perspectives for public health

The findings of this study will contribute to the body of scientific knowledge on HM. This epidemiological data will also contribute to capacity enhancement, treatment, clinical services, advocacy and prevention of HM at KUC. It is timely information and requires a response from the Congolese government and Kinshasa University regarding improvements in early diagnosis, chemotherapy, radiotherapy, hematopoietic stem cell transplantation, supportive care and rehabilitation for HM patients. These remain challenges in DR Congo and sub–Saharan countries [1].

### Strengths and limitations

The novelty of this study was an opportunity to use a rigorous, probabilistic and prospective approach to identifying predictors of HM in Kinshasa, DRC. However, this study was limited because of a lack of cytogenetic methods. The present findings cannot be generalized to the general population of DR Congo.

## Conclusion

These findings highlight the importance of HIV/AIDS, aging, environmental pollutions, the dry season, El Nino and La Nina as related to global warming as determinants of incident hematologic malignancies among black anaemic patients in Kinshasa, DR Congo. While significant findings have been documented on the HV/AIDS epidemic, concerted efforts of individuals, government and donor agencies are needed to address environmental pollution in Kinshasa and DRC in view of the current findings. Global leaders should be encouraged to double their efforts on global warming for the overall benefit of mankind and specifically to reduce the scourge of incident HM and other malignancies. Cancer registries in DRC and other African countries will provide more robust database for future researches on haematological malignancies in the light of the findings of this study.

## Abbreviations

ALL: Acute lymphoid leukaemia
AML: Acute myeloid leukaemia
CLL: Chronic lymphoid leukaemia
CML: Chronic myeloid leukaemia
DRC: Democratic Republic of Congo
EDTA: Ethylenediaminetetraacetic acid
FAB: French-American-British Classification System
HM: Hematological malignancies
KUC: Kinshasa University Clinics
MDS: Myelodysplastic syndrome
MGG: May Grunwald Giemsa
MM: Multiple myeloma
NCD: Non-communicable diseases
ONI: Oceanic Nino Index
ROC: Receiver operating characteristic curve
SD: Standard deviation
T2DM: Type 2 diabetes mellitus
